# Intranasal dexamethasone: a new clinical trial for the control of inflammation and neuroinflammation in COVID-19 patients

**DOI:** 10.1186/s13063-022-06075-5

**Published:** 2022-02-14

**Authors:** Graciela Cárdenas, María Chávez-Canales, Ana María Espinosa, Antonio Jordán-Ríos, Daniel Anica Malagon, Manlio Fabio Márquez Murillo, Laura Victoria Torres Araujo, Ricardo Leopoldo Barajas Campos, Rosa María Wong-Chew, Luis Esteban Ramirez González, Karent Ibet Cresencio, Enrique García Velázquez, Mariana Rodriguez de la Cerda, Yoana Leyva, Joselin Hernández-Ruiz, María Luisa Hernández-Medel, Mireya León-Hernández, Karen Medina Quero, Anahí Sánchez Monciváis, Sergio Hernández Díaz, Ignacia Rosalia Zeron Martínez, Adriana Martínez-Cuazitl, Iván Noé Martínez Salazar, Eduardo Beltrán Sarmiento, Aldo Figueroa Peña, Patricia Saraí Hernández, Rafel Ignacio Aguilar Reynoso, Daniela Murillo Reyes, Luis Rodrigo del Río Ambriz, Rogelio Antonio Alfaro Bonilla, Jocelyn Cruz, Leonor Huerta, Nora Alma Fierro, Marisela Hernández, Mayra Pérez-Tapia, Gabriela Meneses, Erick Espíndola-Arriaga, Gabriela Rosas, Alberto Chinney, Sergio Rosales Mendoza, Juan Alberto Hernández-Aceves, Jaquelynne Cervantes-Torres, Anai Fuentes Rodríguez, Roxana Olguin Alor, Sandra Ortega Francisco, Evelyn Alvarez Salazar, Hugo Besedovsky, Marta C. Romano, Raúl J. Bobes, Helgi Jung, Gloria Soldevila, Juan López-Alvarenga, Gladis Fragoso, Juan Pedro Laclette, Edda Sciutto

**Affiliations:** 1 Instituto Nacional de Neurología y Neurocirugía (INNN), Av. Insurgentes Sur 3877, La Fama, Tlalpan, 14269 Mexico, Mexico; 2 Unidad de Investigación UNAM-INC, Instituto Nacional de Cardiología Ignacio Chávez and Instituto de Investigaciones Biomédicas, Universidad Nacional Autónoma de México, Juan Badiano No. 1, Col. Sección XVI, Tlalpan, 14080 Mexico, Mexico; 3 Clinical Pharmacology Unit, Hospital General de México Dr. Eduardo Liceaga, Dr. Balmis 148, Doctores, Cuauhtémoc, 06720 Mexico, Mexico; 4 Instituto Nacional de Cardiología Ignacio Chávez, Juan Badiano 1, Belisario Domínguez Secc 16, Tlalpan, 14080 Mexico City, CDMX, Mexico; 5 Facultad de Medicina, Universidad Nacional Autónoma de México, 04510 Mexico, Mexico; 6 Unidad Temporal COVID-19, Centro Citibanamex, Avenida del Conscripto 311, Lomas de Sotelo, 11200 Miguel Hidalgo, CDMX, Mexico; 7 Hospital Militar, Secretaría de la Defensa Nacional, Periférico Blvrd Manuel Ávila Camacho s/n, Militar, Miguel Hidalgo, 11200 Mexico City, CDMX, Mexico; 8 Departamento de Inmunología, Instituto de Investigaciones Biomédicas, Universidad Nacional Autónoma de México, Circuito escolar s/n, 04510 Mexico, Mexico; 9 Unidad de Desarrollo e Investigación en Bioprocesos, Escuela Nacional de Ciencias Biológicas del Instituto Politécnico Nacional, Prolongación de Carpio y Plan de Ayala S/N, Col. Casco de Santo Tomas, Del. Miguel Hidalgo, 11340 Mexico, Mexico; 10 Instituto de Diagnóstico y Referencia Epidemiológicos Dr. Manuel Martínez Báez, Francisco de Miranda 177, Lomas de Plateros, Álvaro Obregón, 01480 Mexico, Mexico; 11 Facultad de Medicina, Universidad Autónoma del Estado de Morelos, Avenida Universidad No. 1001, Chamilpa, 62209 Cuernavaca, Morelos Mexico; 12 Universidad Autónoma de San Luis Potosí, Álvaro Obregón 64, Col. Centro, C.P. 78000, San Luis Potosí, S.L.P, Mexico; 13 Laboratorio Nacional de Citometría de Flujo, Instituto de Investigaciones Biomédicas, UNAM, 04510 Mexico, Mexico; 14 Institute of Physiology and Pathophysiology, Emil-Mannkopff-Straße 2 u, 35037 Marburg, Germany; 15 Departamento de Fisiología, Biofísica y Neurociencias, Centro de Investigación y Estudios Avanzados del Instituto Politécnico Nacional, Av. Instituto Politécnico Nacional 2508, San Pedro Zacatenco, Gustavo A. Madero, 07360 Mexico, Mexico; 16 Facultad de Química, Universidad Nacional Autónoma de México, 04510 Mexico, Mexico; 17 School of Medicine, University of Texas Rio Grande Valley – UTRGV, 1201 W University Dr, Edinburg, TX 78539 USA

**Keywords:** Dexamethasone, Intranasal administration, Inflammation, Neuroinflammation, COVID-19

## Abstract

**Background:**

By end December of 2021, COVID-19 has infected around 276 million individuals and caused over 5 million deaths worldwide. Infection results in dysregulated systemic inflammation, multi-organ dysfunction, and critical illness. Cells of the central nervous system are also affected, triggering an uncontrolled neuroinflammatory response. Low doses of glucocorticoids, administered orally or intravenously, reduce mortality among moderate and severe COVID-19 patients. However, low doses administered by these routes do not reach therapeutic levels in the CNS. In contrast, intranasally administered dexamethasone can result in therapeutic doses in the CNS even at low doses.

**Methods:**

This is an approved open-label, multicenter, randomized controlled trial to compare the effectiveness of intranasal versus intravenous dexamethasone administered in low doses to moderate and severe COVID-19 adult patients. The protocol is conducted in five health institutions in Mexico City. A total of 120 patients will be randomized into two groups (intravenous vs. intranasal) at a 1:1 ratio. Both groups will be treated with the corresponding dexamethasone scheme for 10 days. The primary outcome of the study will be clinical improvement, defined as a statistically significant reduction in the NEWS-2 score of patients with intranasal versus intravenous dexamethasone administration. The secondary outcome will be the reduction in mortality during hospitalization.

**Conclusions:**

This protocol is currently in progress to improve the efficacy of the standard therapeutic dexamethasone regimen for moderate and severe COVID-19 patients.

**Trial registration:**

ClinicalTrials.govNCT04513184. Registered November 12, 2020. Approved by La Comisión Federal para la Protección contra Riesgos Sanitarios (COFEPRIS) with identification number DI/20/407/04/36. People are currently being recruited.

**Graphical abstract:**

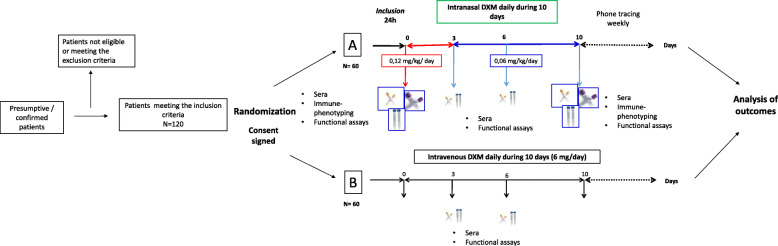

## Highlights

- REVIVAL is a multicenter, open-label, randomized, controlled study to compare the standard low doses of intravenous dexamethasone with weight-adjusted low doses of intranasal dexamethasone.
- Intranasal dexamethasone can reach the respiratory tract more effectively than intravenous administration.
- Intranasal dexamethasone can reach the central nervous system in therapeutic concentrations, even at low doses.
- REVIVAL aims to reduce central failures and sequelae by controlling not only systemic inflammation but also neuroinflammation.

## Background

So far, the outbreak of COVID-19 has infected around 276 million individuals and caused over 5 million deaths worldwide (https://coronavirus.jhu.edu/map.html), with a current global case-fatality ratio of 2%. The most affected geographic region is the Americas, with a case-fatality ratio of 2.4%.

Several factors predict a poor outcome for COVID-19 patients. These include comorbidities (diabetes, hypertension, obesity) and aging, which are normally accompanied by a dysregulated inflammatory response [1]. Other relevant factors include SARS-CoV-2 neurotropism/neuroinvasive [2–9] as viral RNA was found in the brain of patients who deceased from severe acute respiratory syndrome due to COVID-19 infection [10–12]. Likewise, evidence of astrocytic activation and neuronal damage was reported in severe COVID-19 patients with elevated plasmatic levels of glial fibrillary acidic protein and neurofilament light polypeptide [13]. Other authors have shown extensive infection of astrocytes [14] and neurons in 2D and 3D cultures [15, 16]. The infection of cells of the central nervous system (CNS) results in the expression of pathogen-associated molecular patterns (PAMPs) and danger-associated molecular patterns (DAMPs) that trigger a neuroinflammatory response. The exacerbated systemic inflammation, combined with the consequent breakdown of the blood-brain barrier, induces the migration of cells and peripheral inflammatory mediators into the brain. Together, these factors intensify and sustain neuroinflammation, which, added to peripheral damage, may contribute to multi-organ dysfunction and death [10, 12].

### Natural history of SARS-CoV-2 infection

**Fig. 1:**
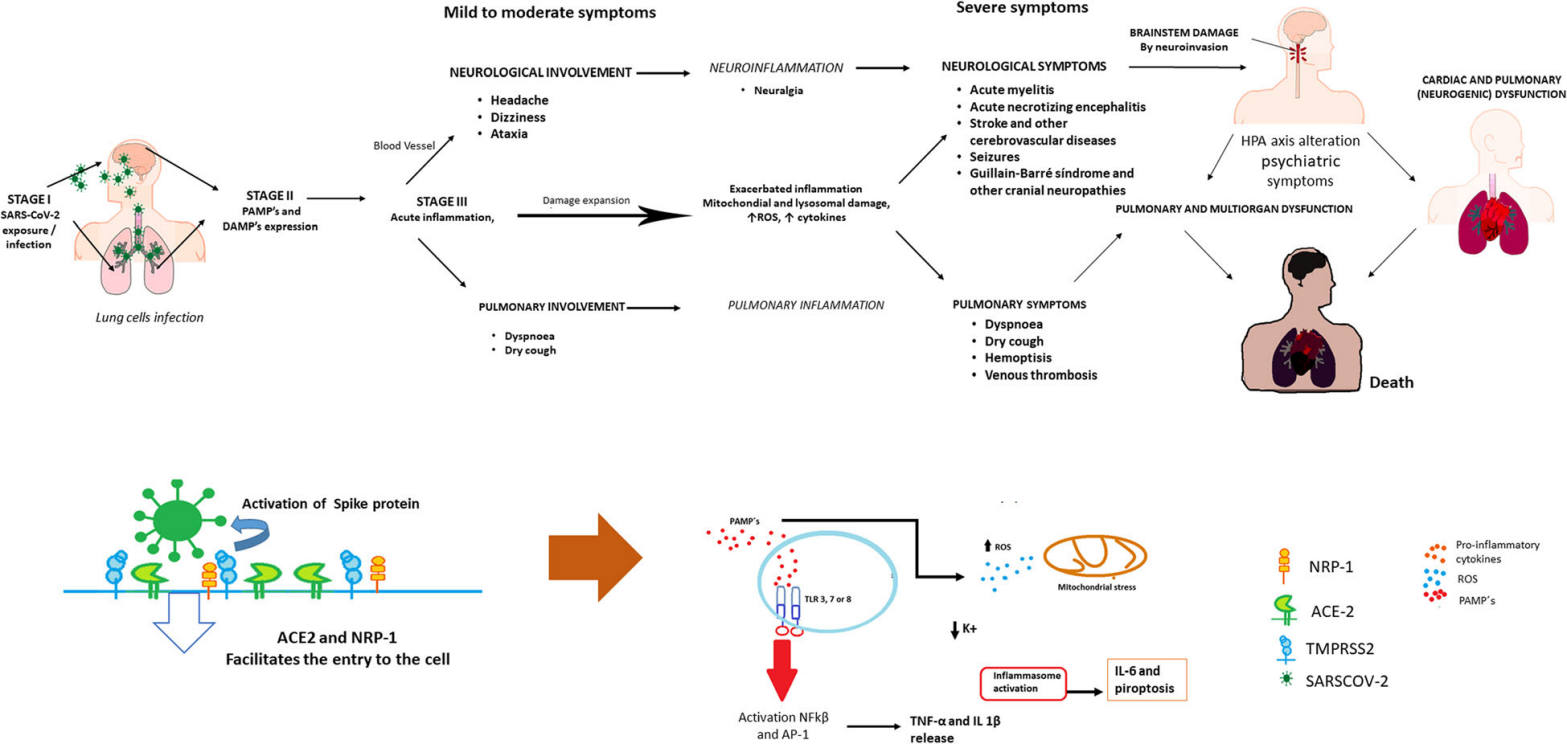
Inflammatory phenomena associated with SARS-CoV-2 infection and its neurological and respiratory manifestations. The SARS-CoV-2 virus mainly enters the respiratory tract and reaches the lungs through direct ventilation and the CNS through the olfactory and trigeminal nerves. The entry of the virus is facilitated by NRP-1, ACE2 receptors, and protein S activation by TMPRSS2. In the CNS, the virus infects neurons, glial cells, and endothelial cells, increasing the permeability of the BBB. This may cause cerebral edema, intracranial hypertension, and neuroinflammation. If the viral infection continues, the damage spreads throughout the body, causing heart and systemic failure. This damage is associated with increased neuroinflammation directed by microglia and oligodendrocytes, causing damage to the brain stem and dysfunction of the heart and lungs. The exacerbated inflammation and intravascular coagulation induce respiratory arrest, possibly leading to the patient’s death. The inflammation is triggered by viral components (PAMPS) that activate TLR3, 7, and 8 receptors on the cell surface. Consequently, there is an increased production of pro-inflammatory cytokines (TNFα and IL 1β) and ROS, which can modify the P2X7 receptor in the brain and activate the inflammasome by the decrease of K^+.^ The activation of the inflammasome increases the production of IL-6 and pyroptosis. This diagram is based on the knowledge at the time of writing the manuscript

A clinical staging system for SARS-CoV-2 infection has been proposed. It involves four stages: early infection (Stage I, mild), pulmonary involvement without hypoxia (Stage IIa, moderate), or with hypoxia (Stage IIb), and systemic hyperinflammation (Stage III) [17] (Fig. 1).

After exposure to SARS-CoV-2, the virus enters the host through the nasal cavity and respiratory airway. Early infection (Stage I) courses with mild and non-specific symptoms (fever, malaise, and asthenia); upon this prodromic phase, the virus binds to its target receptors ACE2, TMPRSS2 [18, 19], and NRP-1, the most recently discovered target [20, 21]. These receptors are highly present in several tissues, including the olfactory neuroepithelium, although to a lesser extent in the sensory olfactory neurons, and the lung [19–22]. Thus, the infection can be established in the lungs (Stage II), leading to viral pneumonia, cough, and fever with or without hypoxia. In the lung, SARS-CoV-2 PAMPs will be recognized by endosomal TLR3, TLR7, and TLR8, and cytosolic RIG-I-like receptors [23]. The virus can also reach the CNS through the olfactory and trigeminal nerve terminals. Once in the CNS, it can infect and damage the endothelium, pericytes, and neural cells that express ACE2 and NRP-1 receptors [20, 21], promoting neuroinflammation (Fig. 1). CNS viral involvement is related to headache, dizziness, and ataxia, but infection also may progress to the whole brain, including the brainstem [5, 6]. In a minority of infected patients, the disease progresses to Stage III, coursing with a hyperinflammatory syndrome. This syndrome is characterized by the sustained production of pro-inflammatory cytokines (including IL-1β and TNFα) and reactive oxygen species (ROS), mitochondrial and lysosomal damage, and the hyperactivation of P2X7 receptors. These processes induce the activation of the inflammasome, which increases IL-6 and leads to pyroptosis. Consequently, the dissemination of viral antigens and RNA into the circulation establishes a persistent inflammatory cycle. Immune complexes can also be generated and deposited in target organs [23–25]. During this phase, sustained neuroinflammation may exacerbate the neuronal injury, spreading damage and contributing to central respiratory failure, ultimately resulting in multi-organ dysfunction [17].

A crucial strategy to treat COVID-19 patients is controlling neural and systemic inflammation. For this purpose, it is essential to consider how viruses invade the human organism. The intranasal route is the most frequent; it allows direct access to both respiratory and central nervous systems through neural pathways [5, 15–18]. Coronaviruses, including SARS-CoV-2, can infect brainstem neurons associated with cardio-respiratory control; thus, pulmonary function is also altered at the central level [5, 26–29]. COVID-19 neurological clinical symptoms, particularly nausea, vomiting, and dysgeusia, seem to involve the dorsal vagal complex (DVC) and the *nucleus tractus solitarius* (NTS), linked to the control of several autonomic functions [26]. The NTS is also a well-known target of neuro-immune activation [30]. Its ascending projections reach the hypothalamic paraventricular nucleus, involved in the activation of the HPA axis, and the rostral ventrolateral medulla (RVM), which controls respiratory and cardiovascular functions [31].

The viral infection in the respiratory and central nervous systems promotes the expression of PAMPs and DAMPs. These signals trigger the inflammasome and oxidative stress [23, 32]. Later during infection, the inflammatory response may become dysregulated, extending the initial damage caused by the infection.

### Adrenal insufficiency in SARS-CoV-2 infection

Critically ill patients affected by different pathologies frequently show adrenal insufficiency, which may increase morbidity and mortality [33, 34]. COVID-19 might affect the hypothalamic-pituitary-adrenal (HPA) axis as well. Hypothalamic and hypophyseal tissues express ACE2 and can therefore be viral targets [35]. The virus may directly damage the hypothalamus and the pituitary, leading to hypothalamic-pituitary dysfunctions.

Since the SARS outbreak of 2003, autopsy studies have demonstrated that coronaviruses affect the HPA axis and promote vasculitis in several organs, including adrenal glands; in particular, adrenal cortical cells undergo degeneration and necrosis [36]. Although the full long-term spectrum of COVID-19 endocrine manifestations is still unclear, several endocrine alterations have been reported in SARS survivors. These include hypocortisolism, hypothyroidism, and low levels of dehydroepiandrosterone, suggesting a transient hypothalamic-pituitary dysfunction [37]. Recently, an Arabian study including 28 patients reported the adrenal response to an acute COVID-19 infection; the median level of morning cortisol was 196 (31–587) nmol/L, and the median level of ACTH was 18.5 (4–38 ng/L). Interestingly, patients with severe disease had lower cortisol and ACTH [38]. In addition, other autopsy studies have found edema, neuronal degeneration, and evidence of viral genome in the hypothalamus [39]. Thus, in the presence of subacute thyroiditis or adrenal insufficiency, corticosteroid therapy should help by reducing high amounts of thyroid hormones and replace adrenal function, improving the evolution of these patients regardless of the route of administration.

### Rationale

Dexamethasone sodium phosphate (ALIN, injectable solution. Chinoin Laboratory) is a highly soluble glucocorticoid with a pH = 7–8.5 that does not harm the nasal mucosa. This synthetic steroid is an anti-inflammatory and immunomodulatory drug that inhibits platelet activation, prostaglandin and leukotriene synthesis, and coagulation by regulating transcriptional factors like NF-κB and AP-1 [40–42]. Furthermore, DXM exerts important neuroprotective effects such as rescuing neurovascular integrity during neuroinflammation [43].

### Dexamethasone: a potent anti-inflammatory drug

Considering that the complications of COVID-19 result from exacerbated peripheral and neural inflammation derived from the so-called cytokine storms, at least three key points have been addressed in the use of DXM for the treatment of Coronavirus patients: timing, dose, and route of administration. First, the drug should not be applied from the beginning of the infection when inflammation favors the control of viral replication and the establishment of an adaptive immune response that serves to control the infection. A low dose of DXM (6 mg per patient for 10 days) has quickly and effectively controlled pulmonary inflammation with minimal negative side effects [44]. In addition, the intranasal route would allow direct access of DXM to the CNS through the olfactory and trigeminus nerves, thereby controlling the sustained neuroinflammation provoked by damage to infected astrocytes, neurons, and microglia. Therefore, cardiac and central respiratory failure could be diminished in COVID-19 patients, avoiding fatalities.

In experimental models, it is well known that drugs administered intranasally usually grant higher bioavailability in the CNS compared with similar doses administered intravenously, since intranasal drugs bypass the BBB and hepatic degradation [45–48]. In addition, intranasal DXM might control inflammation by arriving directly to the respiratory system more effectively and quickly than the intravenous route [46–49]. DXM prevents the binding of ACE2 to the spike protein of SARS-CoV-2 and can also bind to LYS353, an active residue of the receptor-binding domain (RBD) [50]. Moreover, DMX reduces ACE2 expression in several cell types by suppressing type I interferon expression [51]; it can also downregulate neutrophil extracellular traps (NETs), possibly through regulating Toll-like receptors [52]. Hyperinflammation is related to high levels of NETs and neutrophilia, which, in turn, predicts thrombosis and poorer outcomes in acute respiratory distress syndrome (ARDS) [53, 54].

## Methods

### Trial design

The “REVIVAL” trial is an interventional, phase 2, multicenter, open-label, randomized controlled study in adult patients with confirmed COVID-19 diagnosis, designed to evaluate the efficacy (superiority) of low doses of intranasal DXM compared to intravenous administration (allocation ratio 1:1) in patients of five COVID-19 referral centers in Mexico City.

### Settings and trial sponsor

This clinical trial is being conducted at the five Health Institutions in Mexico City: “Hospital General de México Dr. Eduardo Liceaga,” “Instituto Nacional de Neurología y Neurocirugía Manuel Velasco Suárez,” “Instituto Nacional de Cardiología Ignacio Chavez,” “COVID-19 unit at Citibanamex,” and “Hospital Central Militar.”

The Hospital General de México Dr. Eduardo Liceaga (HGMEL) is the trial sponsor investigator which is in charge of initiating, administering, and monitoring the current clinical trial. The hospital address is Dr. Balmis 148, Doctores, Cuauhtemoc, CP06720, Mexico City and the telephone is (+ 52)5527892000. The current protocol is already approved by HGMEL (#DI/20/407/043).

When recruitment of all participants be completed, the trial sponsor investigator will also participate in the interpretation of data, writing the paper, and the decision to submit the report for publication.

### Eligibility criteria

**Fig. 2:**
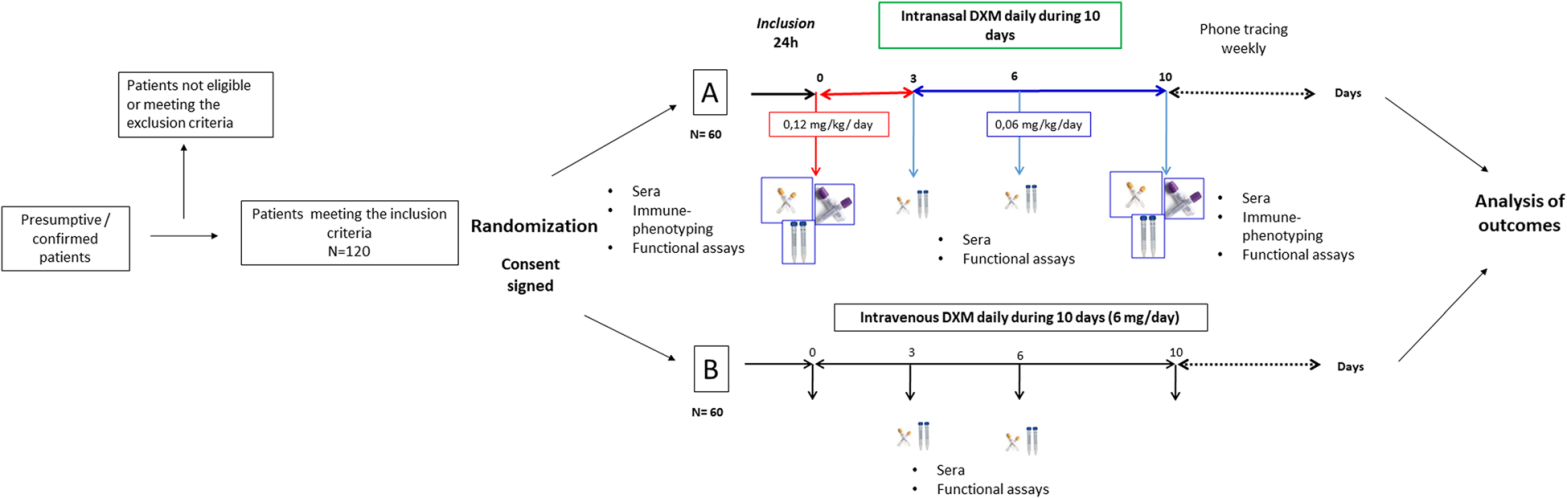
Outline of the REVIVAL trial clinical protocol. Initially, patients will be informed about the clinical trial; if they accept and sign the consent, they will be randomized using the Sealed envelope® software. Group A will receive intranasal DXM; Group B will receive intravenous DXM. Both groups will be sampled on days 0, 3, 6, and 10 post-treatment to collect sera and nasopharyngeal swabs. Patients will be monitored throughout the study. The results will be tested for statistical differences between groups

Inclusion criteria comprise patients of both sexes (non-pregnant female), aged from 18 to 90 years old, with presumptive SARS-CoV-2 infection and more than 5 days of clinical evolution, with moderate to severe symptoms requiring oxygen support or high flux mechanical ventilation (NEWS-2 ≥ 5), and abnormal CT-chest scan CO-RADS > 3. Patients should be diagnosed with atypical pneumonia, confirmed by chest images and oxygen saturation (SpO_2_) lower than 93% in ambient air or a ratio of the partial pressure of oxygen and the fraction of inspired oxygen (PaO_2_: FiO_2_) equal to or lower than 300 mmHg, and a positive RT- PCR SARS-CoV-2 test. These patients will be allocated into the experimental group or the control group in a 1:1 ratio (two arms) (Fig. 2) according to the randomization.

Exclusion criteria include patients with an RT-PCR SARS-CoV-2 negative test; previously receiving GCs at high doses by oral or intravenous administration; severely immunosuppressed as in AIDS, pregnancy, and autoimmune diseases; those who have received outpatient treatment with steroids for more than 72 h before hospital admission; those older than 90 years; with DXM allergy; risk for glaucoma; or recurrent respiratory diseases. Patients receiving other monoclonal antibody-based treatments such as tocilizumab are also excluded. No other concomitant treatments are prohibited for patient eligibility.

Elimination criteria include voluntary withdrawing, lack of informed consent letter, or imminent risk of death within 48 h.

### Interventions

#### Groups and comparators

The study will be carried out in two groups; group A (experimental) will receive intranasal DXM, and group B (Control) will receive intravenous DXM (Fig. 2). This experimental design is based on previously reported data indicating that intranasal administration can reach the brain and bloodstream quickly and efficiently [46–49]. Group A will receive intranasal DXM daily, at the dose of 0.12 mg/kg for the first 3 days, followed by 0.06 mg/kg for 7 days. Group B will receive 6 mg of intravenous DXM daily. In each hospital, the Pharmacovigilance staff will collect the adverse event information, for any (untoward o abnormal) medical manifestation, symptom, or disease, whether or not related with the drug treatment in each patient throughout the study.

#### Procedures

After randomization, a Case Report Format (CRF) (printed or electronic) for each patient will be filled daily by the medical staff and completed at the end of treatment or fatal outcome, whatever occurs first. Blood and saliva samples will be collected every third day during the whole treatment to perform ancillary tests such as SARS-CoV-2 viral load, functional immunological assessment (lymphocyte cytometry, cytokine/chemokine profile), and cortisol levels, among other analyses.

All biological samples will be each 48 h at every hospital center participant, transported by a specialized service and concentrated at Instituto de Investigaciones Biomédicas (UNAM) to be managed and stored at − 70 °C until use. As mentioned in the Informed Consent, all samples will be used for the next 3 years. Additional biological samples may be taken at each hospital center when necessary, according to the treating physicians and will not be part of this protocol.

All personal data and medical information of the patients will be treated in a strictly confidential way. Only the lead investigator and the hospital coordinator investigators will have access to this information.

#### Participants

The sample includes 120 adult patients of both sexes between 18 and 90 years of age, with moderate and severe forms of COVID-19. Participants are recruited from the five participant hospitals. Medical teams at each participating Hospital Center will be led by a responsible clinician who will obtain the informed consent letter. All eligible patients or a responsible family member (in the case that the patient cannot sign) receives an informed consent letter from the lead clinician, where the characteristics of the procedure are detailed. A medical team at each hospital will oversee the enrollment of participants according to a randomization scheme. A clinical monitor will supervise the data generation, protocol implementation, and appropriate patient enrollment.

#### Sample size and randomization

The analysts of the statistic team calculated the size with EPIDAT version 3.1.2 software, using the option “Sample size and surveillance curves” with an estimated 50% increase in the proportion of patients free of mechanical ventilation [intranasal DXM 70% vs. intravenous DXM 45%]. This value was estimated using the data of the COVID-19 patients registered in Mexican hospitals with a confidence of 95%, power of 80%, and proportion of losses of 10%. A sample size of 60 patients per group was obtained with these parameters. The randomization will be made with Sealed Envelope software. This software is freely available from https://www.sealedenvelope.com/simple-randomiser/v1/lists [Accessed May 5, 2020]. This study is a multicenter randomized controlled trial (Fig. 2).

#### Confidentiality

Each patient who agrees to participate in the protocol will be assigned an identification number that will be used throughout the procedure. This code distinguishes the hospital of origin and the patient’s identification number. All the information collected during the procedure will be confidential (following the data privacy statement found in the informed consent letter) and used only for research purposes and follow-up of adverse effects.

#### Outcomes

The expected primary outcome is clinical improvement, defined as a two-point improvement in the ordinal scale regarding the initial NEWS-2 score. This is an adapted score recommended by the World Health Organization (WHO) and the National Institute for Health and Care Excellence (NICE) to facilitate the early recognition and escalation of deteriorating patients [55, 56]. Although this score is commonly adopted for triage, it can also be used for continuous assessment, particularly in predicting intrahospital mortality. In this context and based on previous experimental data regarding the sustained effect of intranasal dexamethasone, we decided to evaluate the improvement in the initial NEWS-2 score as the first outcome.

The secondary expected outcome includes a reduction in mortality that will be examined during treatment (after randomization), a reduction of the time required for mechanical ventilation, the length of patient’s stay in the hospital, 30-day post-discharge mortality, and intrahospital complications. Viral load, the immune-inflammatory profile, and other physiological parameters will also be evaluated before and after treatment (see above).

#### Data collection and management

After acceptance and signature of the informed consent letter, patients will be randomized, saliva and nasopharyngeal samples will be taken to measure the viral load, and treatment will begin as indicated in Fig. 2. The patient’s clinical history will be based on the initial results and physical inspection. Blood and saliva samples will be collected before the start of DXM treatment and at days 3, 6, and 10 within the treatment period. All statistical analyses will be performed by our team of specialists.

The samples will be sent for specialized analysis following standardized operating procedures (SOP’s). All patient data, including clinical history and laboratory data, will be collected daily and managed using REDCap (Research Electronic Data Capture System) tools hosted at Instituto de Investigaciones Biomédicas, UNAM [57, 58].

#### Plans to promote participant retention and complete follow-up

All participants will receive specialized medical care, including clinical, neurological, and neuropsychological studies. These evaluations will be carried out 1, 3, 6, and 12 months after COVID-19 to monitor the evolution of the disease. The participants that present any functional post-COVID decline will receive medical treatment and neurorehabilitation.

Likewise, patients who present an adverse effect or health problem during their participation in this study or derivate to it upon hospitalization will receive all necessary treatment and care until their resolution in the “Hospital General Hospital de Mexico Dr. Eduardo Liceaga.” In addition, patients will be monitored every 3 months for 1 year after the study. During the study, other immunomodulatory drugs such as colchicine, GM-CSF inhibitors, IVIG, interferons, interleukins inhibitors, and kinase inhibitors can be used under the responsibility of the physician.

#### Data monitoring committee

A multidisciplinary group of independent experts will be on charge to assess the progress, safety data, and if needed, critical efficacy endpoints of this study.

#### Data management

The information collected during the procedure will be documented physically and digitally in an exact and precise manner. Researchers will use complete patient reports along with the molecular and immunological tests to analyze the outcomes. The information collected will be treated as confidential, and only the global results will be published without showing the patients’ names. In case data are required, the information can be requested from the researchers with valid reasons.

#### Statistical analysis

Descriptive statistics using mean (standard deviation) or frequency (percentage) will be used following the dimension scale of the variable. An initial comparison between the hospital will define relevant differences among them. Since healthcare conditions are variable in each hospital included in this study, we will analyze the results with a multilevel mixed longitudinal model. The outcome analysis for NEWS-2 components (i.e., respiration rate, oxygen saturation, systolic blood pressure, pulse rate) will be done using the last observation carried forward (LOCF) in those subjects with at least two observations, this analysis will avoid over-optimistic estimates of efficacy. Other efficacy variables like biochemistry or cell counts will be analyzed without ITT due to the susceptibility to type II error.

Biochemical variables and cell counts will be aggregated using principal component analysis and use the scores if the Kaiser-Meyer-Olkin (KMO) is greater than 0.6 and sphericity *p* < 0.0001. The scores using varimax rotation will be used as dependent variables if the method is suitable for our data. The longitudinal mixed model can be described as:

$$ {y}_{ij}={X}_{ij}\beta +{u}_i+{v}_{j(i)}+{\epsilon}_{ij} $$

where *j* = 1,..,Ti are nested in 10 time points, within *i* = 1,..,5 hospitals. The Xs correspond to fixed variables like the treatments, age, sex, and weight, among other confounders.

A database in vertical format was reshaped to nest the biochemical, inflammatory markers, cell count adjusted by sex, age, and BMI. We will perform restricted maximum likelihood (REML) estimation in case we obtain balanced samples. However, if we obtain an unbalanced sample, we will use maximum likelihood instead [59]. Because we expect a small sample size from some hospitals, we will make inferences using the Kendward-Roger degrees of freedom method. We will make simultaneous inferences about l linear combinations of fixed factors. Despite the complicated situation where *l* > 1 we will consider:

$$ F=\frac{1}{l}{\left(\hat{\beta}-\beta \right)}^T\ L{\left({L}^T\ {\hat{\varphi}}_AL\right)}^{-1}\ {L}^T\ \left(\hat{\beta}-\beta \right) $$

The internal random structure [${L}^T\ {\hat{\varphi}}_AL$] can change in different settings, but still will be a good approximation. Simulations studies showed adequate performance of modifications with different REML [60]. Finally, the structure of residual error will be analyzed within the lowest-level group. Once recognized, the covariance structure we will use unstructured, Toeplitz, or exponential corrections. This approach will give unbiased approximation even in presence of unbalanced samples.

The residuals for our mixed models will be considered by the source of present variability: The marginal residuals will predict the marginal errors, the conditional residuals, and the best linear unbiased predictor (BLUP) residuals. The conditional residuals will be standardized with:

${\hat{\varepsilon}}_k^{\ast }=\frac{{\hat{\varepsilon}}_k}{\sigma\ \sqrt{p_{kk}}}$ , where *p*_*kk*_ represents the *k*th element of the covariance structure. These residuals are functions of the joint leverage of fixed and random effects.

Subgroup analysis will be based on interaction as secondary analysis. This information will help to identify multiplicative effects from basal conditions like the presence of type 2 diabetes, obesity, hypertension, clinical history IECA medication, or other conditions.

All analysis will describe the number of patients with the outcome of interests, the treatment effect (mainly using eta squared), additive and multiplicative interactions with 95% confidence intervals, and the direction of the interaction (positive or negative) by specific subgroups. The analysis will be performed with Stata version 17.0 [StataCorp LLC College Station TX]. A statistical difference with *P* < 0.05 will be considered significant.

#### Interim analysis

This analysis will be performed by an independent expert team to examine some relevant data as baseline, safety outcome, and efficacy outcome data to consider ending the study if no trend is observed

## Conclusions

Intranasal DXM at low doses could be a more effective therapeutic option to control peripheral and central inflammation during ARDS in severe and critical forms of SARS-CoV-2 infection. In addition, it could stabilize the activity of the HPA axis upon this severe stress condition. Although low-dose systemically administered DXM is beneficial for COVID-19 patients, it cannot reach effective therapeutic concentrations in the CNS to control neuroinflammation. In contrast, intranasal administration of DXM is highly effective in controlling neuroinflammation, as demonstrated in experimental inflammation models [41–44]. Therefore, in the REVIVAL trial clinical protocol, we propose boosting the effect of low-dose DXM treatment through an intranasal route of administration. This route will allow reaching the CNS at therapeutic doses that may effectively reduce the morbidity and mortality in severe or critical COVID-19 patients, further than that reported in the RECOVERY trial.

Low doses of intranasal DXM are currently being tested (clinicaltrials.gov id: NCT04513184) in a randomized study in hospitalized COVID-19 patients (moderate and severe forms). The clinical evolution and respiratory parameters of the patients receiving intranasal DXM (experimental treatment) are compared with those of patients receiving 6 mg of intravenous DXM, the currently recommended treatment (https://www.covid19treatmentguidelines.nih.gov/). Considering the prevalence of metabolic syndrome and obesity in Mexico, the therapeutic scheme has been weight-adjusted. A DXM dose of 0.12 mg/kg is administered for 3 days, followed by a dose of 0.06 mg/kg for 7 days. If the current approach yields fewer adverse effects and does reach the CNS to control neuroinflammation as we hypothesized, there will be direct interest to extend this protocol to several COVID hospitals of the National Health System in Mexico. In addition, increasing the initial sample size (preliminary results) will be required to publish the study and share it with the International scientific community.

## Data Availability

Data and materials are not available at this moment, because the work being considered is the first approach to a clinical trial currently started. When the study will be completed, the dataset obtained and analyzed will be available from the corresponding author only by reasonable request.

## Abbreviations

COVID-19: Coronavirus disease 19
CNS: Central nervous system
GCs: Glucocorticoids
NEWS-2: National Early Warning Score-2
COFEPRIS: Comisión Federal para la Protección contra Riesgos Sanitarios
SARS-CoV-2: Severe acute respiratory syndrome coronavirus 2
RNA: Ribonucleic acid
GFAP: Glial fibrillary acidic protein
NfL: Neurofilament light protein
PAMPs: Pathogen-associated molecular patterns
DAMPs: Damage-associated molecular patterns
ACE2: Angiotensin-Converting Enzyme 2
TMPRSS2: Transmembrane Serine Protease 2
NRP-1: Neuropilin -1
TLR: Toll-like receptor
IL-1β: Interleukin-1β
TNFα: Tumor Necrosis Factor α
ROS: Reactive oxygen species
P2X7: Purinergic Receptor P2X7
DVC: Dorsal vagal complex
NTS: Nucleus Tractus solitary
HPA: Hypothalamic-pituitary-adrenal
RVM: Rostral ventrolateral medulla
ACTH: Adrenocorticotrophic hormone
NF-κB: Nuclear Factor Kappa-Light-Chain-Enhancer of activated B cells
AP-1: Activating Protein-1
ATP: Adenosine Triphosphate
NLRP3: NLR Family Pyrin Domain Containing 3
DXM: Dexamethasone
BBB: Blood-brain barrier
RBD: Receptor-binding domain
NETs: Neutrophils extracelullar traps
ARDS: Acute respiratory distress syndrome
CO-RADS: Coronavirus-19 Reporting and Data System
RT-PCR: Reverse transcription polymerase chain reaction
AIDS: Acquired immunodecficiency syndrome
CRF: Case report format
HGMEL: Hospital General de México Dr. Eduardo Liceaga
